# Supplementary material for: Genome-Wide Identification, Diversification, and Expression Analysis of Lectin Receptor-Like Kinase (LecRLK) Gene Family in Cucumber under Biotic Stress
Source: Int J Mol Sci. 2021 Jun 19;22(12):6585. doi: 10.3390/ijms22126585 (PMC8234520; doi:10.3390/ijms22126585)
Supplement: Supplementary file 1 [file ijms-22-06585-s001.zip › IJMS_Supplementary Figures.pdf]

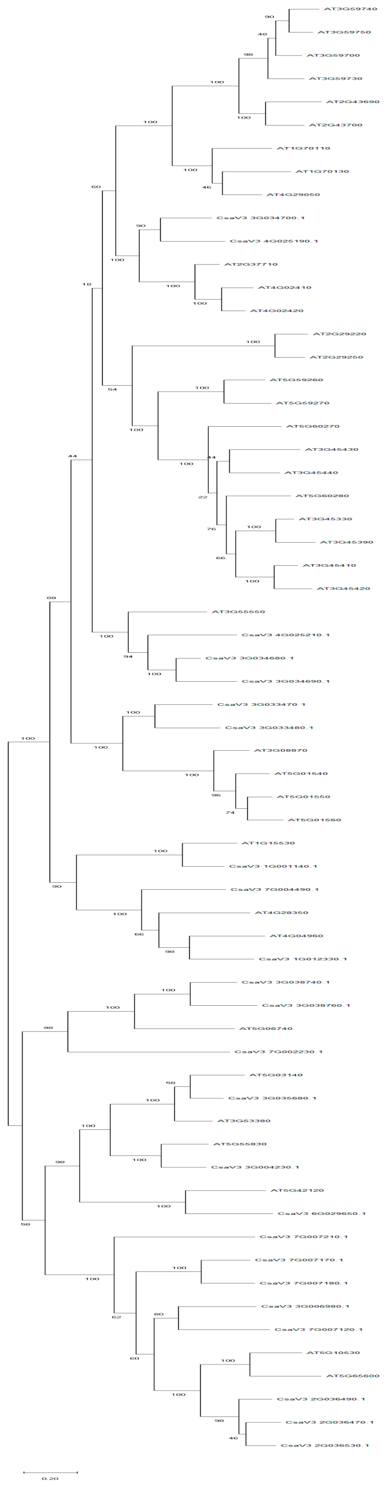

Supplementary Figure S1. Phylogenetic analysis of L-type LecRLK proteins between cucumber and Arabidopsis. The phylogenetic tree was constructed by MEGA (7.0) software using the Maxi-mum Likelihood (ML) method with 1000 bootstrap values.

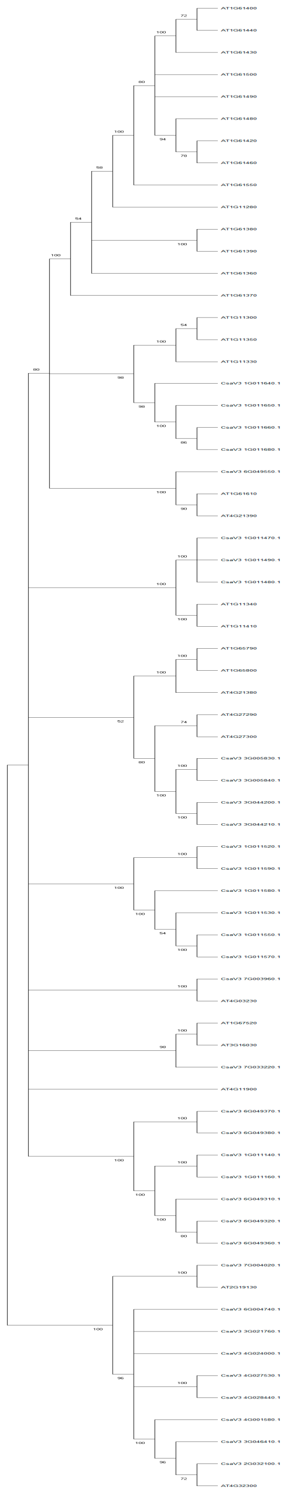

Supplementary Figure S1. Phylogenetic analysis of G-type LecRLK proteins between cucumber and Arabidopsis. The phylogenetic tree was constructed by MEGA (7.0) software using the Maxi-mum Likelihood (ML) method with 1000 bootstrap values

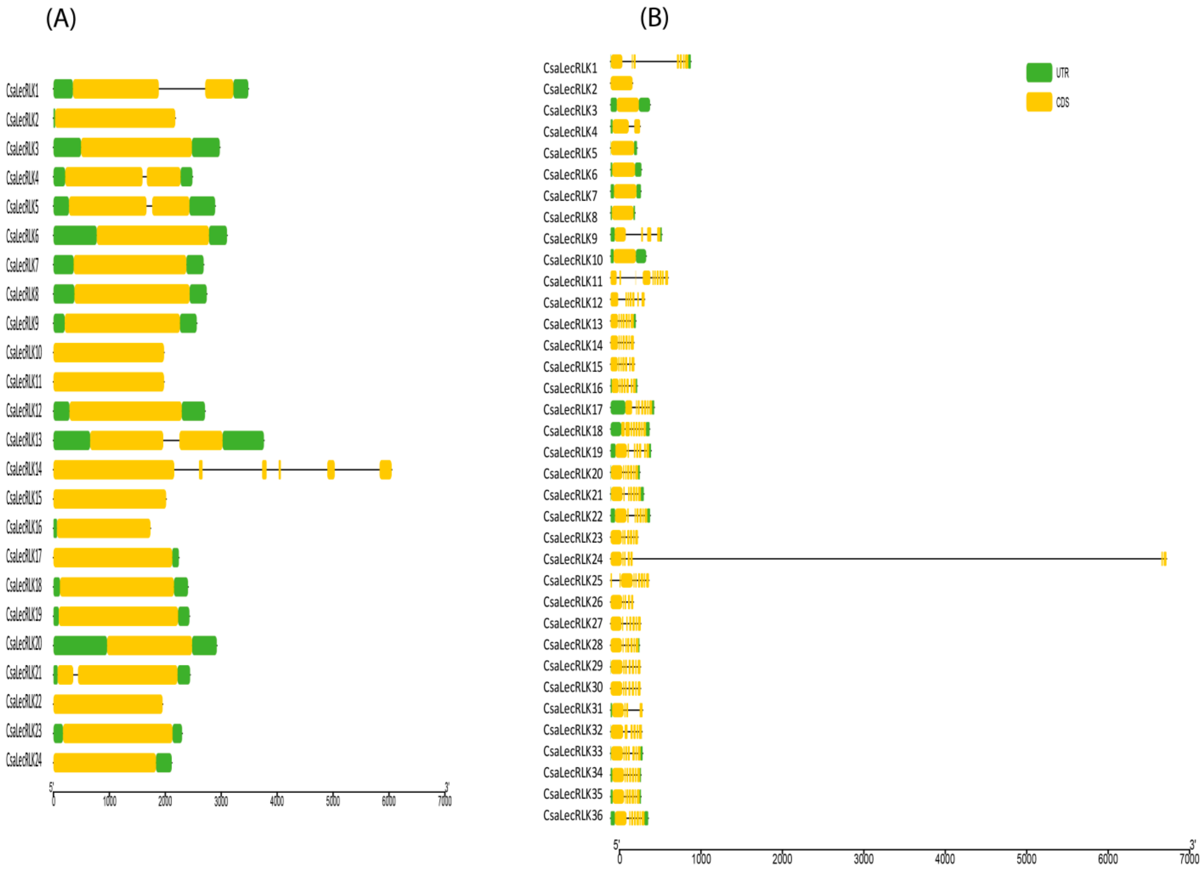

Supplementary Figure S3. The gene structure of coding sequences (CDS) and untranslated regions (UTRs) of (A) L-type and (B) G-type *LecRLK* genes in cucumber illustrated using TB tools.

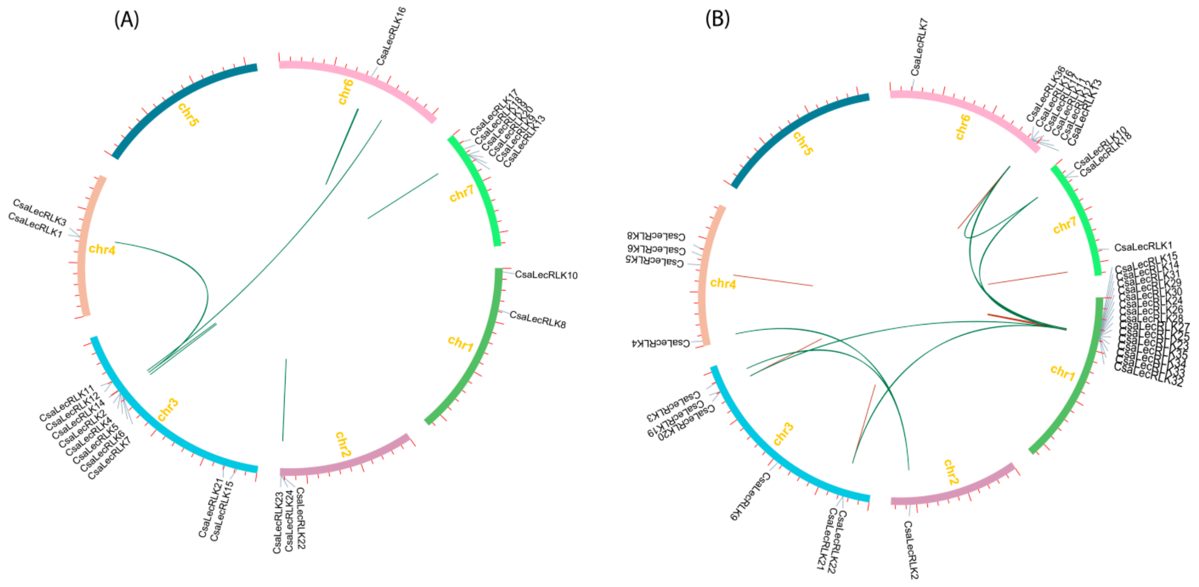

Supplementary Figure S4. The collinearity analysis showing higher conservation among (A) L-type LecRLK proteins as compared to (B) G-type LecRLK proteins.

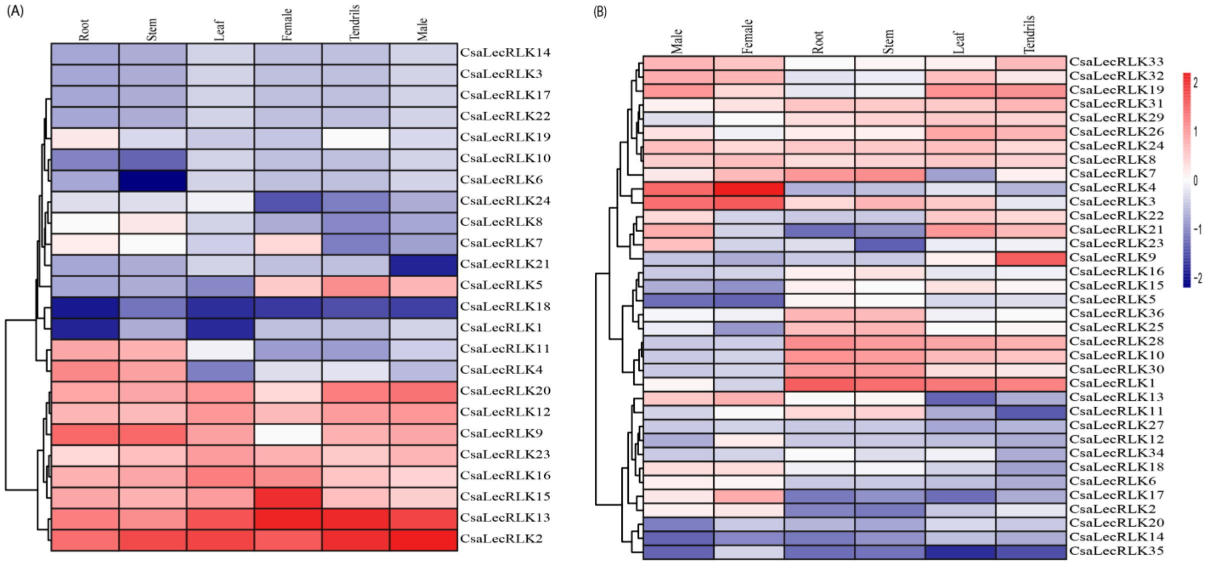

Supplementary Figure S5. RNA-seq findings of (A) L-type LecRLK and (B) G-type LecRLK genes in different tissues of cucumber. The heatmap was generated based on the Log_2 value using the Rstudio program (A package of R, USA)".
