# Supplementary figures and images for: Genome-Wide Identification, Diversification, and Expression Analysis of Lectin Receptor-Like Kinase (LecRLK) Gene Family in Cucumber under Biotic Stress
Source: Int J Mol Sci. 2021 Jun 19;22(12):6585. doi: 10.3390/ijms22126585 (PMC8234520; doi:10.3390/ijms22126585)

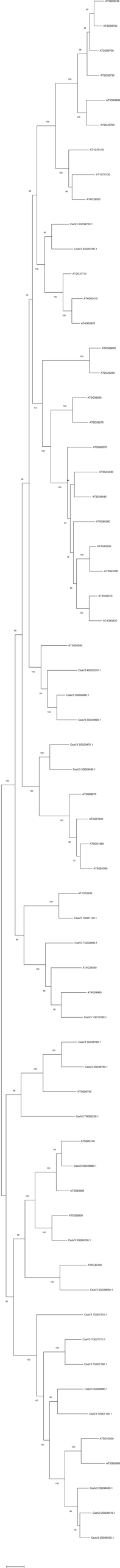

Supplement: Supplementary file 1 [file ijms-22-06585-s001.zip › Figure S1.pdf]

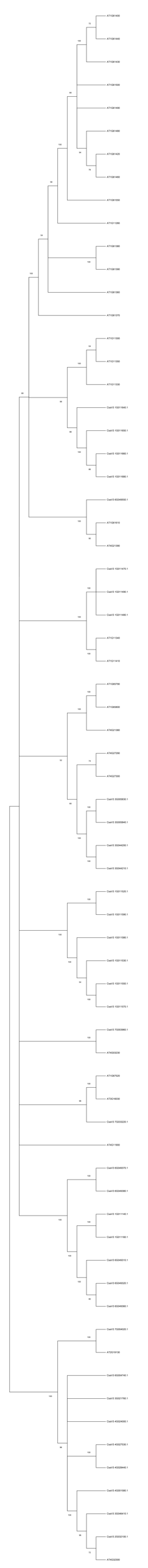

Supplement: Supplementary file 1 [file ijms-22-06585-s001.zip › Figure S2.pdf]
